# Chyluria: non-enhanced MR lymphography

**DOI:** 10.1186/s13244-023-01461-2

**Published:** 2023-07-05

**Authors:** Alexandre Sabbah, Cedi Koumako, Sanaâ El Mouhadi, Amal Ali, Lise Minssen, Quentin Vanderbecq, Lionel Arrivé

**Affiliations:** Department of Radiology, Saint-Antoine Hospital, Assistance Publique – Hôpitaux de Paris (APHP) and Sorbonne University, 184 Rue du Faubourg Saint-Antoine, 75012 Paris, France

**Keywords:** Chyluria, Lymphatics, Filariasis, MR imaging, MR lymphography

## Abstract

Chyluria is an uncommon medical condition resulting from an abnormal communication between the abdominal lymphatic system and the urinary tract, which results in the presence of chyle in the urine, making it appear milky white. Proper diagnosis is demonstrated by the concentration of urinary lipids. Worldwide, chyluria is most commonly associated with the parasite *Wuchereria bancrofti*. However, in Europe and North America, where the condition is rare, non-parasitic etiologies predominate. Identifying the cause and location of the uro-lymphatic communication is essential in guiding therapeutic management, but imaging the lymphatic channels remains a challenge. Magnetic resonance (MR) lymphography, a non-invasive free-breathing 3D high-resolution fast-recovery fast spin-echo sequence similar to that used for 3D MR cholangiopancreatography, may demonstrate the cause and location of an abnormal communication between the lymphatic system and urinary tract. In parasitic causes of chyluria, dilated lymphatics vessels communicating with the lymphatic system are demonstrated. In non-parasitic causes of chyluria channel type lymphatic malformations are the most common. Markedly dilated and dysplastic lymphatic vessels communicating with the urinary tract are demonstrated. In addition, other cystic or channel type lymphatic malformations such as thoracic, soft tissue or bone abnormalities may be observed. This review describes the abdominal lymphatic diseases leading to chyluria and presents the technique and images obtained with non-enhanced MR lymphography to enable radiologists in identifying and classifying uro-lymphatic fistulae.

**Critical relevance statement**: Non-enhanced MR lymphography enables the identification and categorization of uro-lymphatic fistulae.

**Graphical abstract:**

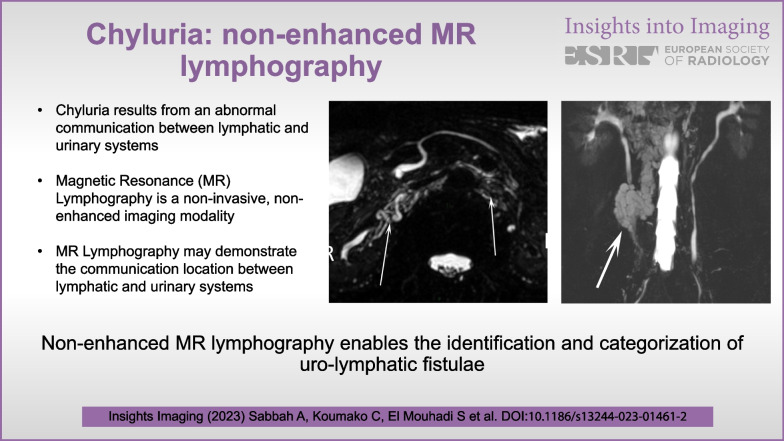

## Key points

1. Chyluria results from an abnormal communication between lymphatic and urinary systems.
2. Magnetic Resonance (MR) Lymphography is a non-invasive, non-enhanced imaging modality.
3. MR Lymphography may demonstrate the communication location between lymphatic and urinary systems.

## Introduction

Chyluria is a rare medical condition defined by the emission of milky white urine due to the presence of chyle, an intestinal lymphatic liquid rich in lipids (chylomicrons), proteins, and lymphocytes that comes from the small intestines [1–4]. Proper diagnosis is based on an increased concentration of urinary lipids [5]. A wide range of other symptoms can be associated with chyluria, such as abdominal or pelvic pain, dysuria, hematuria, or clot colic. Hypoprotidemia with undernutrition, weight loss, and lower limb edema may also be observed. Worldwide, chyluria is most commonly associated with the parasite *Wuchereria bancrofti*, which is prevalent in parts of Asia, especially India, as well as Sub-Saharan Africa. However, in Europe and North America, where the condition is rare, non-parasitic etiologies predominate including lymphatic malformation and other uncommon causes including blunt or penetrating trauma, complications of surgery such as partial nephrectomy or retroperitoneal surgery, non-parasitic infection, malignancy and pregnancy [1–3].

Imaging techniques are principally useful for detecting and identifying the location of the uro-lymphatic fistula. However, due to its anatomical complexity and variations, imaging of the lymphatic system remains difficult and has long been limited to the invasive and constraining technique of conventional lymphography. Non-enhanced MR lymphography based on a free-breathing 3D high-resolution fast-recovery fast spin-echo sequence similar to that used for 3D MR cholangiopancreatography, which focus only on the signal of stationary or low speed fluids, can be used for diagnosis of etiology and uro-lymphatic fistula mapping, helping in decision-making for therapeutic management. Here, we describe the anatomy of the abdominal and pelvic lymphatic system, present the causes and mechanisms of chyluria, and show the imaging features of chyluria on non-enhanced MR lymphography.

## Lymphatic anatomy

The lymphatic system is a network of thin distal blinded vessels, larger collectors, and lymph nodes that play roles in the removal of cellular waste, proteins, and water from the interstitial space, as well as in immune protection and fat absorption from the intestine. Compared to the vascular system, the lymphatic system anatomy is markedly complex and exhibits a lot of variants. Most of the lymphatic channels are very small, and, together with lymph nodes, they create a complex network of interlacing vessels. This complexity and variability are, in part, explained by the embryology of the lymphatic system [6]. Physiologically, the diameter of the lymphatic vessels varies in a regularly alternating way, with the thinner parts corresponding to the lymphatic valves, making the lymphatic circulation unidirectional [7]. They are easily recognized because of the characteristic alternating bands of constriction (lymphatic valves) and dilatation. These characteristics help identify the lymphatic system on MR lymphography.

**Fig. 1:**
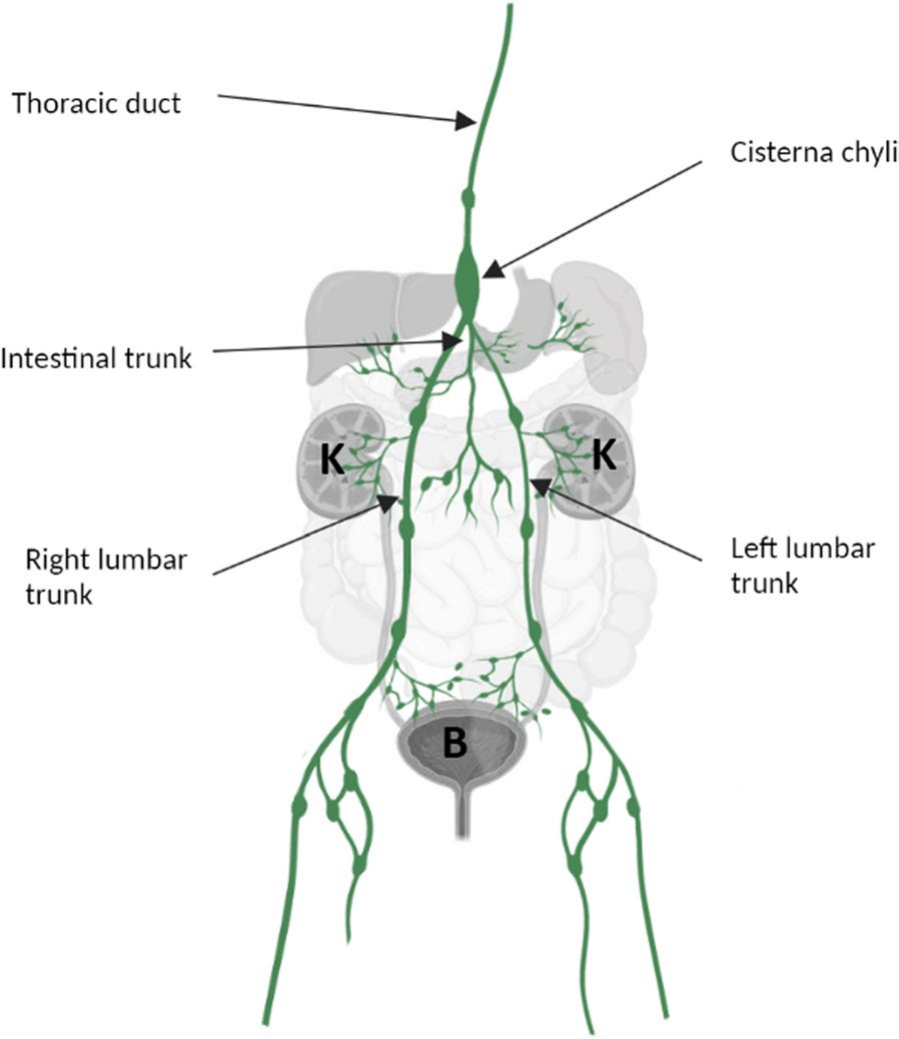
Lymphatic anatomy (K: kidney, B: bladder)

Regarding the abdominal lymphatics, thin mesenteric vessels converge to become one or two mesenteric trunks, which join the intestinal trunk, collecting lymph from the stomach, pancreas, spleen, and liver. The lymph vessels from the kidneys, the deep lymphatics of the abdominal wall, the pelvic organ, and lower limbs converge to form the retroperitoneal lymph trunks (Fig. 1). The retroperitoneal lymph trunks and para-aortic vessels join to form the right and left lumbar trunks, which also converge with the intestinal trunk to constitute the cisterna chyli, just before entering the thorax through the aortic hiatus of the diaphragm and forming the thoracic duct. Typically, the cisterna chyli is a focal saccular lymphatic dilatation located at the L1–L2 level of the retrocrural space, on the right face of the abdominal aorta [6].

Lymph reaches the blood stream as the thoracic duct flows into the junction of the left subclavian and jugular veins. However, this classical anatomy may be subject to multiple variations, such as complex anastomoses of the lumbar lymphatics, resulting in a plexus rather than a cisterna or multiple sacculations of the lymphatic channels. Cisterna chyli size may also be quite different from one patient to another, sometimes making a very large aspect known as the giant cisterna chyli [7]. A consequence of this broad anatomic variation of the cisterna is that some authors prefer to use the descriptive term “abdominal confluence of the lymphatic trunks” rather than cisterna chyli [8].

## Causes of chyluria

Chyluria can be classified into parasitic and non-parasitic causes: 95% of parasitic causes are attributed to Wuchereria bancrofti, with the remaining 5% are secondary to Taenia echinococcus, Taenia nana, Ankylostomiasis, Trichiniasis and the malarial parasite (Table 1). Lymphatic filariasis is, by far, the most prevalent cause of chyluria in the world, reportedly affecting up to 120 million people worldwide. It is endemic in East Asia (especially in the Gangetic belt of India) and Sub-Saharan Africa [9]. Typically, parasitic chyluria affects patients between the ages of 20 and 40 years, but it can occur earlier [2]. Men and women are equally affected. The most common non-parasitic chyluria are related to lymphatic malformations mainly channel type lymphatic malformations. Other less common non-parasitic causes include blunt or penetrating trauma, complications of surgery such as partial nephrectomy or retroperitoneal surgery, infection, malignancy and pregnancy [2].

**Table 1:** Causes of chyluria

Parasitic causes	Non-parasitic causes
Wuchereria Bancrofti (95%)	Lymphatic malformations
Taenia echinococcus	Blunt or penetrating trauma
Taenia nana	Complications of surgery
Ankylostomiasis	Non-parasitic infections
Trichiniasis	Malignant tumors
Malaria	Pregnancy

Chyluria is a rare symptom defined by the emission of milky white urine due to the presence of chyle. Chyle is an intestinal lymphatic liquid that originates from the small intestines (2–4 L/day) when the digested fatty acids, monoglycerides, fat-soluble vitamins, and other nutrients are absorbed [10]. Accordingly, this lymphatic fluid is rich in lipids (chylomicrons), proteins (mostly albumin), and lymphocytes.

A wide range of other symptoms can be associated with chyluria, such as abdominal or pelvic pain, dysuria, urgency and urinary retention secondary to chylous clot, hematuria, or even clot colic. The protein leakage can lead to hypoprotidemia, simulating a nephrotic syndrome with undernutrition, weight loss, and lower limb edema [11]. However, in contrast to nephrotic syndrome, chyluria also presents with hypocholesterolemia and hypotriglyceridemia.

Chyluria implies an abnormal communication between the abdominal lymphatic system and urinary tract. Two theories, obstructive and regurgitation, are mainly used to explain the appearance of a uro-lymphatic fistula and chyluria. When associated with filariasis, the obstructive theory states that it is the obstruction of the lymphatic vessels increasing the lymphatic pressure that causes an opening into the kidney hilum, ureter, or less often the bladder. For chyluria without associated parasitic infection, most often due to a primary lymphatic malformation, the regurgitation theory states that chyluria results from a complex mechanism involving lymphatic parietal hyperpermeability, accumulation of toxic metabolites, and an inflammatory immune reaction, leading to the development of lymphatic varicosities in the vicinity of the urinary tract, resulting in the development of a fistula [12, 13].

Positive diagnosis is performed by quantification of urinary chylomicron, optimally 4 h after a meal rich in fat, as it is the most specific and sensitive test [1]. The threshold of urinary chylomicron used for the diagnosis of chyluria is > 15 mg/dL [2, 14]. The main differential diagnostic is pyuria, for which altered neutrophil polynuclear cells are found in the urine [15].

## Imaging workup of chyluria

Radiological techniques are principally useful for detecting and identifying the location of the uro-lymphatic fistula. However, due to its anatomical complexity and variations, the lymphatic system remains difficult to image and has long been limited to the invasive and constraining technique of conventional lymphography [16].

## Non-enhanced MR lymphography

We show in this pictorial review how non-enhanced MR lymphography, based on a free-breathing 3D high-resolution fast-recovery fast spin-echo sequence similar to that used for 3D MR cholangiopancreatography, which focus only on the signal of stationary or low speed fluids, can be used to localize the uro-lymphatic fistula (Table 2).

**Table 2:** Acquisition parameters of non-enhanced MR lymphography

Field strength	1.5 T
Sequence	3D High-Resolution fast-recovery fast spin-echo (FRFSE)
Plane	Coronal/Axial
TR (ms)	3500–4000
TE (ms)	700–884
Number of averages	1
Flip angle	90°
Matrix acquisition size	512 × 288
FOV (mm)	400 × 400
Number of slices	124–316
Slice thickness (mm)	0.8–1.4
Spacing (mm)	0
Anatomical area	Abdomen
Gating	Free breathing with respiratory gating
Acquisition time (min)	3–5

The main difference between MR lymphography and MR cholangiopancreatography include field of view, plane orientation, and slice thickness. To include retroperitoneal lymphatic vessels and urinary tract, field of view should be larger than that used for MR cholangiopancreatography. As an alternative to coronal plane, MR lymphography may be performed with axial source images which precisely demonstrate both anterior and posterior lymphatic vessels. Because of the small size of lymphatic vessels, very thin section source images (millimetric or submillimetric) are used. Scan time varies from 3 to 5 min, depending on the number of source images. Post-processing of the data is performed to obtain maximum intensity projection (MIP) images. Imaging at 3 Tesla results in improved signal-to-noise ratio that allows for the improvement of spatial resolution. However, motion artefact, susceptibility effect, and local field inhomogeneity distributed throughout the full set of images may decrease image quality.

MR imaging presents several advantages, including very high and unique quality contrast between soft tissues, multiplanar capability, and a non-invasive and non-irradiating character, and only a few formal contraindications (i.e., severe claustrophobia) [17]. Recent technical advances (gradient performance, pulse sequence, and coil improvement) have also made it possible to significantly reduce acquisition times, particularly due to fast spin echo and single shot fast spin echo sequences based on a very long echo train [18].

Because of the spontaneous high contrast, one can analyze the lymphatic vessels and urinary tract with a very high signal intensity. However, lymphatic vessels should be differentiated from other high signal intensity structures on MR lymphography. The bowel fluid signal is easily removed by administering pineapple juice or a diluted paramagnetic contrast material. It is simple to distinguish the lymphatic duct system from the urinary system. Renal pelvis, ureter and bladder have characteristic shapes while lymphatic vessels are recognized because of the characteristic alternating bands of constriction (lymphatic valves) and dilatation. Furthermore signal intensity of lymphatic vessels is usually lower than that of urinary tract possibly due to the high protein concentration of lymph. Under these conditions, it is possible to precisely locate the uro-lymphatic fistula. It is also feasible to determine the pattern of uro-lymphatic fistula (Table 3). In parasite-related chyluria obstruction of the lymphatic vessels by the parasite results in a marked dilatation of lymphatic vessels that may communicate with urinary tract. On the other hand, dysplastic vessels are not observed. In addition, it is not uncommon to demonstrate several locations of potential uro-lymphatic fistulae with non-enhanced MR lymphography. However, under these conditions, it is not possible to determine which of the fistulae are active.

**Table 3:** MR lymphography features observed in chyluria

	Frequency	MR features	Associated abnormalities
Parasitic causes	Very common in endemic area (Asia, Sub-Saharan Africa)	Dilatation of lymphatic vessels without dysplastic vessels	Lower limbs lymphedema
Lymphatic malformations	Common in Europe and North America	Marked dilatation of lymphatic vessels with dysplastic vessels	Multiple lymphatic abnormalities including soft tissues, thoracic, bone lesions
Other causes	Uncommon	Dilatation of lymphatic vessels without dysplastic vessels	None

Lymphatic malformations caused by abnormal development of the lymphatic system are rare somatic diseases. They present as fluid-filled cisterns, so-called cystic lymphatic malformation (previously called lymphangioma) or fluid-filled channels, so-called channel type lymphatic malformation (previously called lymphangiectasis).

Cystic lymphatic malformations are the most common congenital lymphatic anomalies. They present as lesions of variable size classified into macrocystic, microcystic, or mixed cystic lymphatic malformations. Macrocystic lymphatic malformations are large fluid-filled cavities, while microcystic and mixed cystic LMs contain small cysts. Lymphatic malformations can be found anywhere in the body, from extremities to the abdominal or thoracic cavities.

Channel type lymphatic malformations are characterized by dilation, malformation, and dysfunction of the abdominal or thoracic lymphatic vessels, leading to impaired lymph drainage and leakage of lymph (or chyle) into the abdominal cavity (chylous ascites), thoracic cavity (chylothorax) or urinary tract (chyluria). In chyluria, channel type lymphatic malformations are the most commonly observed lymphatic abnormalities. They may be associated with cystic lymphatic malformations especially in case of complex lymphatic anomalies.

Generalized lymphatic anomaly (GLA) is characterized by diffuse or multicentric lymphatic disorders in multiple organs, including the bones, liver, spleen, lungs, and soft tissues [10].

The large field of view of non-enhanced MR lymphography allowed us to image some of these associated abnormalities such as thoracic, soft tissues, or bone locations.

**Fig. 2:**
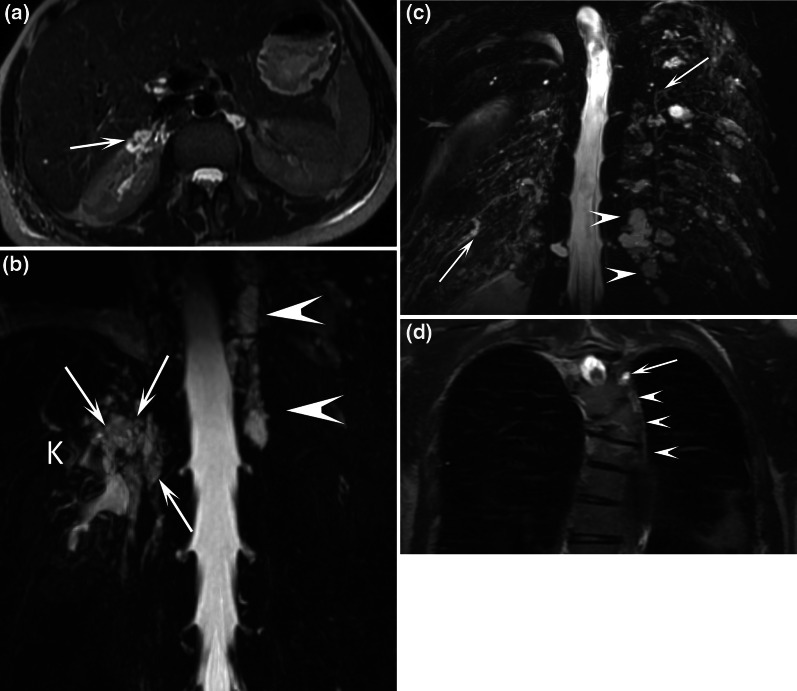
38-year-old female patient with chyluria, chylothorax, and Gorham-Stout disease. Axial T2-weighted MR image (**a**) demonstrated dilated and enlarged lymphatic vessels in contact with the right kidney (arrow). Coronal MR lymphography with MIP reconstruction (**b**) demonstrated channel type lymphatic malformation (arrows) in contact with the right kidney (K). Mixed channel and cystic lymphatic malformation (arrowheads) replaced the thoracic duct. At the thoracic level, coronal MR lymphography with MIP reconstruction **c** demonstrated multiple dilated intercostal lymphatic vessels (arrows) with several cystic lymphatic malformations (arrowheads). Coronal T2-weighted MR image (**d**) demonstrated vertebral fractures, with a hyperintense signal within three vertebrae related to progressive osteolysis caused by Gorham-Stout disease (arrowheads). A cystic lymphatic malformation in contact with the vertebra was also demonstrated (arrow)

**Fig. 3:**
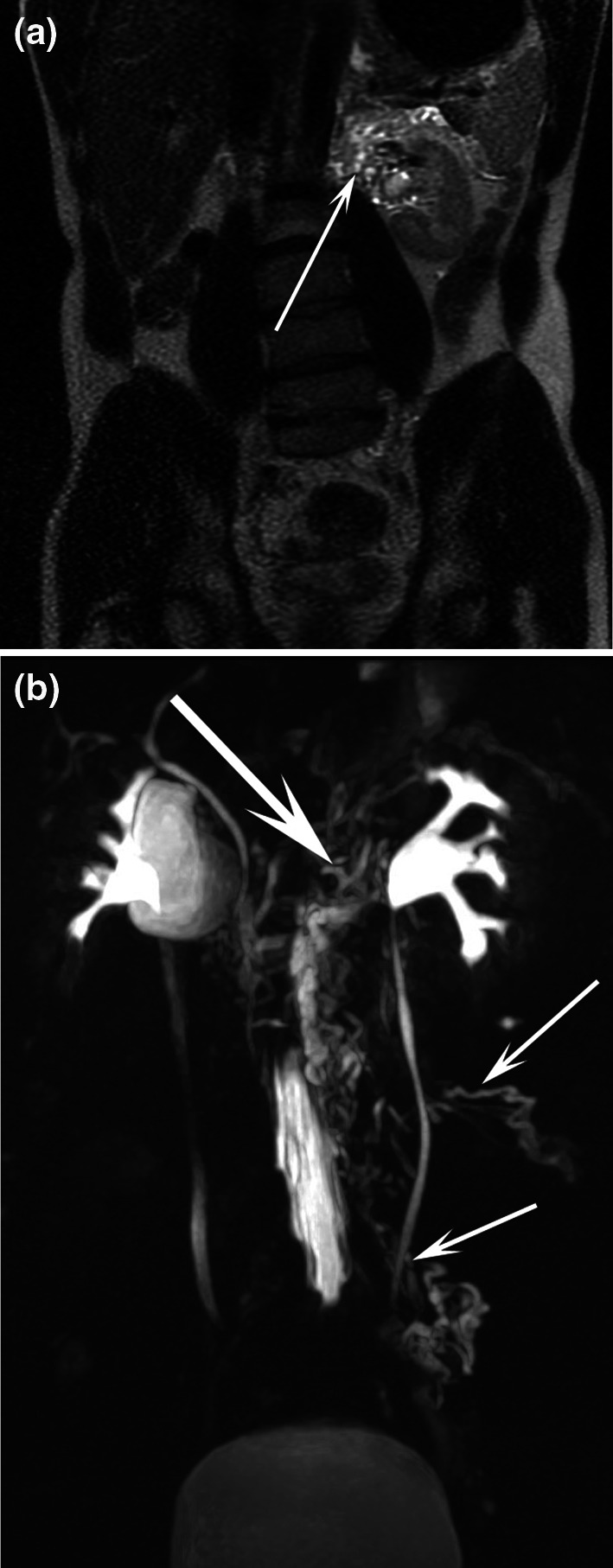
37-year-old male patient with chyluria. Coronal T2-weighted MR image **a** demonstrated abnormal lymphatic vessels (arrow) in touch with the left renal pelvis, which was better seen by (**b**) coronal MR lymphography with MIP reconstruction. Dilated lymphatic vessels close to the left ureter (short arrows) were also demonstrated

**Fig. 4:**
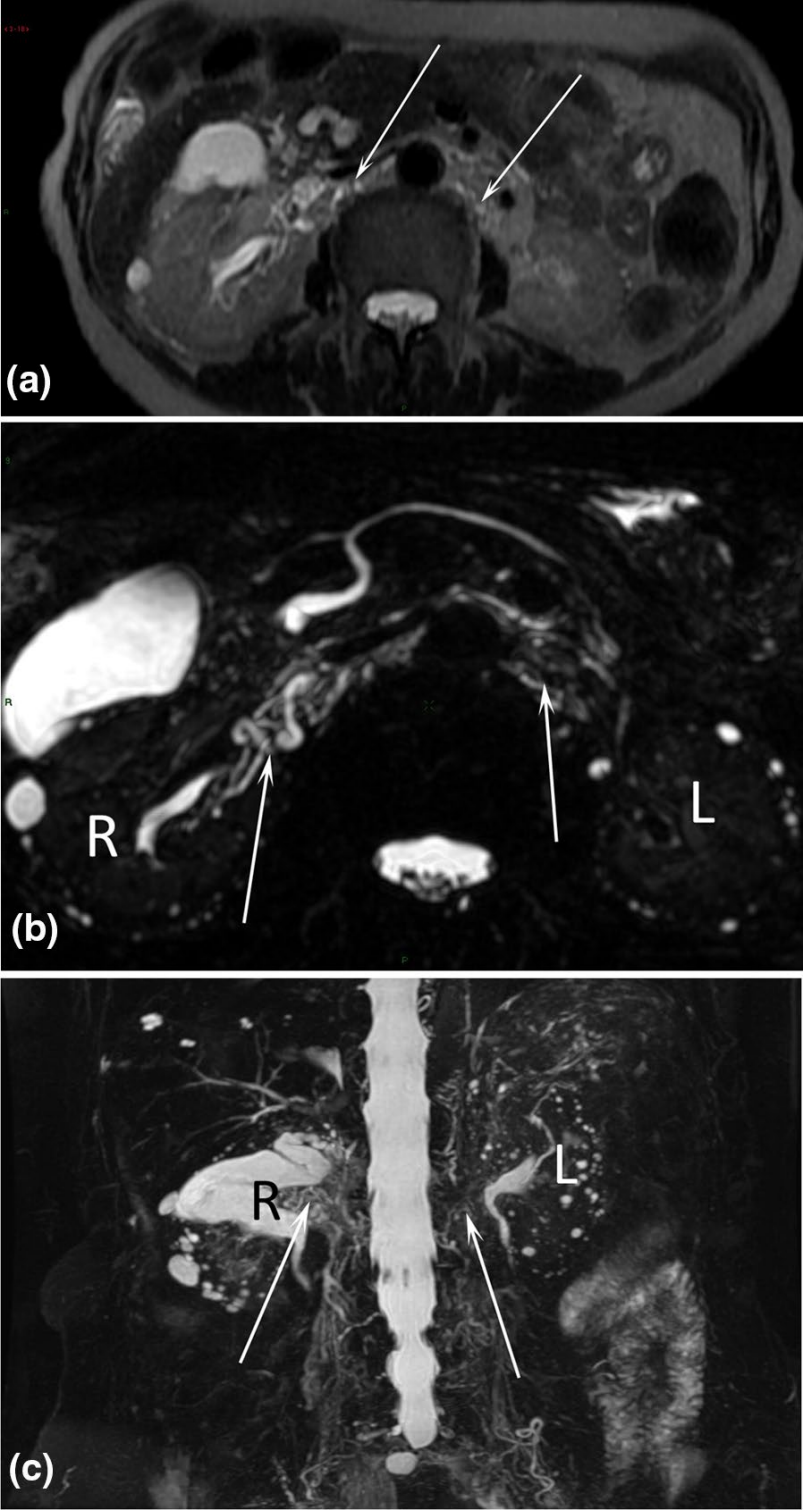
83-year-old female patient with chyluria secondary to filariasis. Axial T2-weighted MR image **a** demonstrated dilated and enlarged lymphatic vessels prevailing on the right side in contact with the right and left kidneys (arrows). The dilated lymphatic vessels (arrows) were better highlighted on MR lymphography with MIP reconstruction in the axial (**b**) and coronal (**c**) planes. *R* right kidney, *L* left kidney

**Fig. 5:**
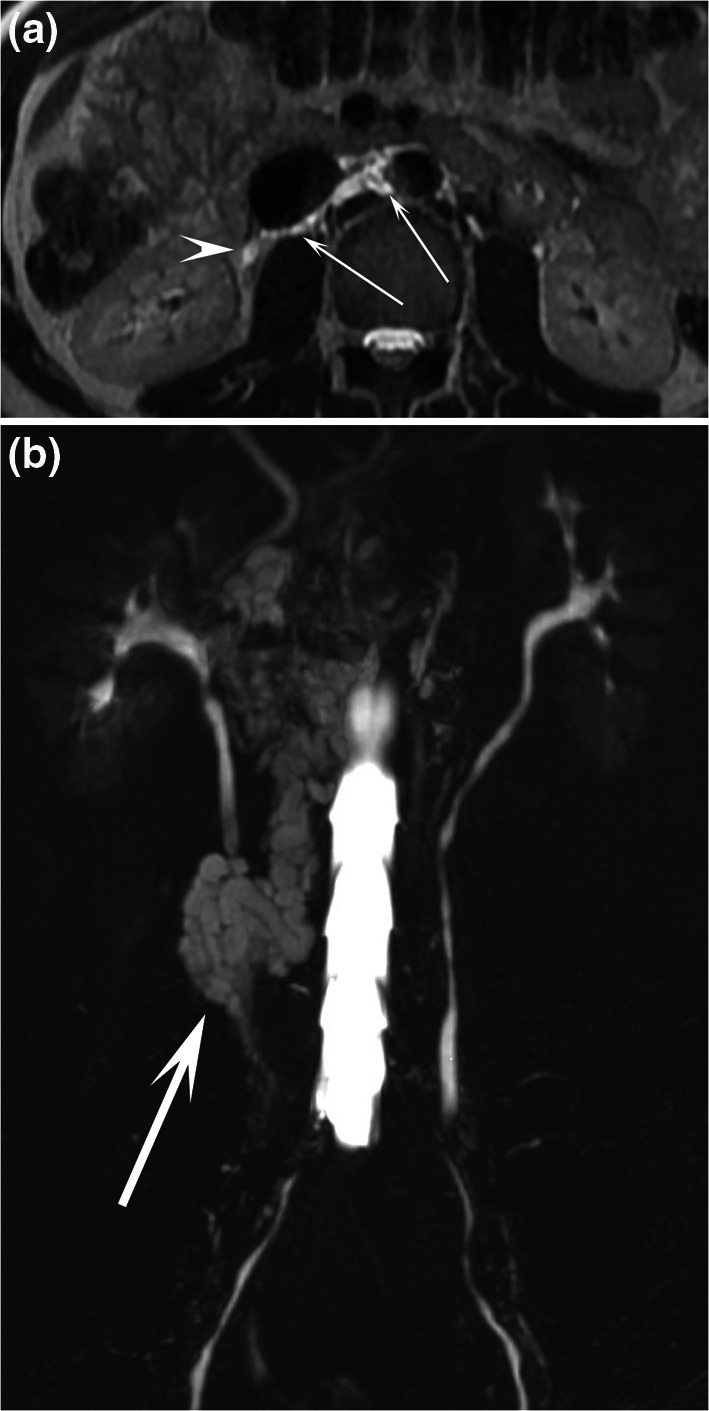
35-year-old male patient with chyluria. Axial T2-weighted MR image **a** demonstrated dilatation of the retroperitoneal lymphatic vessels prevailing on the right side (arrows) and in touch with the right ureter (arrowhead). Coronal MR lymphography with MIP reconstruction (**b**) demonstrated a channel type lymphatic malformation (arrows) in contact with the right renal pelvis and right ureter

**Fig. 6:**
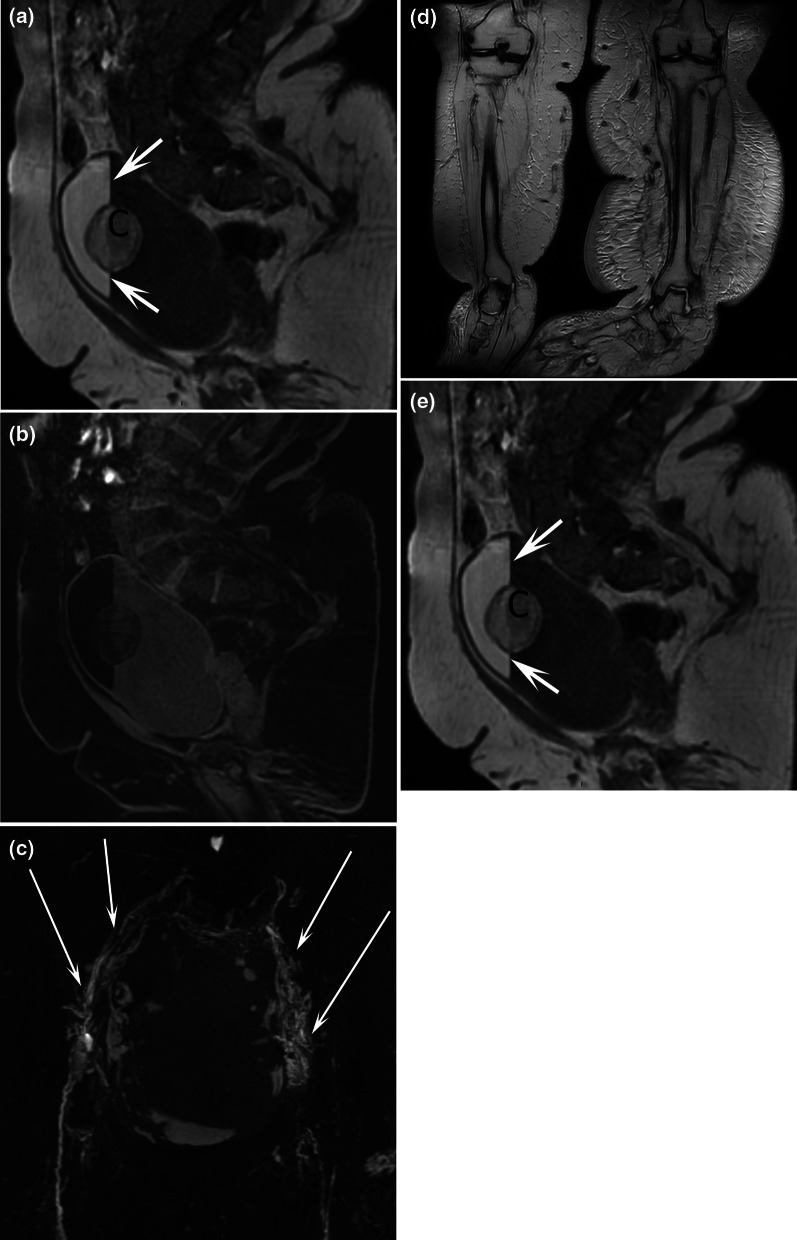
44-year-old female patient with chyluria. Sagittal T2-weighted MR image (**a**) and T1-weighted MR image with fat saturation (**b**) demonstrated an intravesical fat-fluid level (arrows) and chylous clot (C). Coronal MR lymphography with MIP reconstruction **c** demonstrated a channel type lymphatic malformation (arrows) in touch with both faces of the bladder. Coronal MR lymphography with MIP reconstruction (**d**) demonstrated bilateral lower limb lymphedema (L) that was more severe on the left side with dilatation of lymphatic vessels (arrows). Corresponding coronal T2-weighted MR image (**e**) showed bilateral congenital deformities of the ankles and feet

**Fig. 7:**
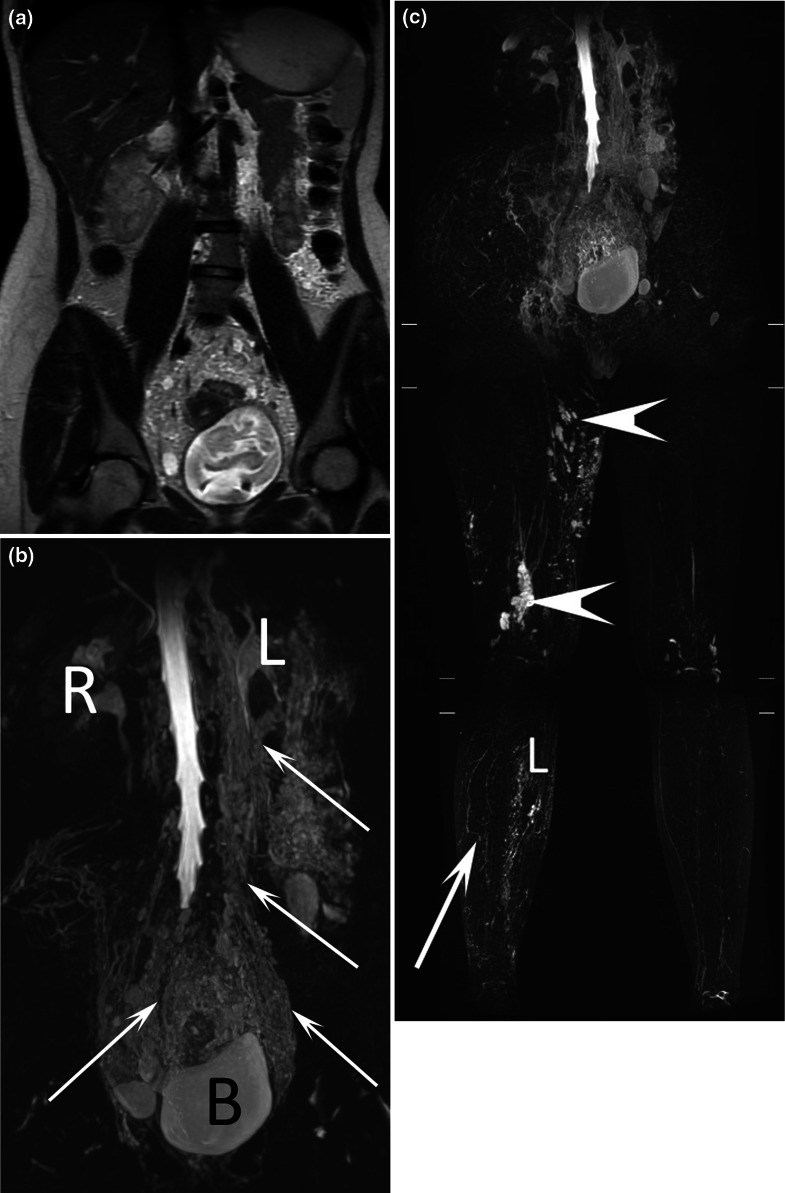
17-year-old female patient with chyluria and vaginal chylous discharge. Coronal T2-weighted MR image **a** demonstrated abnormal lymphatic vessels in the abdomen, pelvis, and retroperitoneum, which were better seen with (**b**) coronal MR lymphography with MIP reconstruction. Dilated lymphatic vessels (arrows) close to the bladder, ureters, colon, and uterine cervix were also demonstrated. *R* right kidney, *L* left kidney. This patient also presented with right lower limb lymphedema (L) and cystic lymphatic malformations (arrows) on coronal MR lymphography with MIP reconstruction (**c**)

**Fig. 8:**
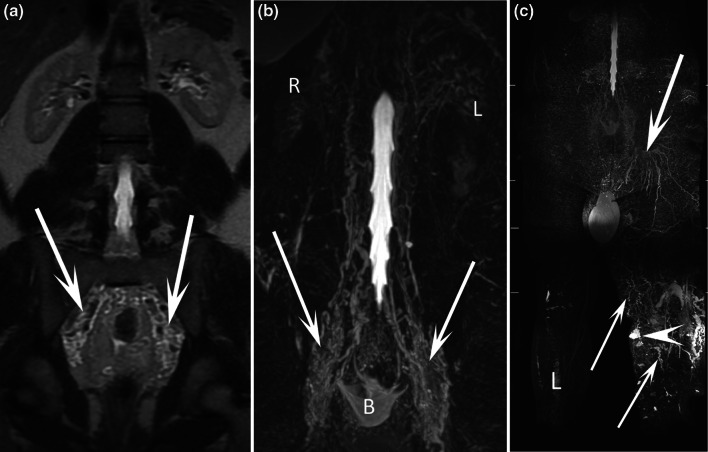
17-year-old male patient with chyluria, hydrocele, and bilateral lymphedema of the lower limbs. Coronal T2-weighted MR image (**a**) and coronal MR lymphography with MIP reconstruction **b** demonstrated widespread dilated lymphatic vessel development along the urinary tract from the retroperitoneum to the bladder (arrows). *R* right kidney, *L* left kidney. Coronal MR lymphography with MIP reconstruction (**c**) demonstrated major lymphatic dysplasia at the root of the left lower limb (arrows) and the presence of several cystic lymphatic malformations (arrowhead) of the soft tissues

We classify the chyluria according to the location of the presumed uro-lymphatic communication: renal communication (Fig. 2), renal pelvis communication (Figs. 3, 4), ureteral communication (Fig. 5), or bladder communication (Fig. 6). Several patients had filariasis, but the majority were found to have lymphatic malformations. We also show different patterns of associated lymphatic malformations (Figs. 2, 7, 8).

## Other imaging techniques

Contrast-enhanced MR lymphangiography uses a contrast medium composed of paramagnetic macromolecule that is injected subcutaneously and preferentially captured by the lymphatic vessels. This allows observation of the lymphatic drainage from a functional point of view, with a better spatial resolution than lymphoscintigraphy [19].

The introduction of intranodal MR lymphangiography has led to enhanced success rates and reduced procedure times when compared to traditional fluoroscopic pedal lymphangiography [20]. In dynamic contrast-enhanced MR lymphangiography, the contrast agent is injected through the inguinal lymph nodes. Sequential acquisition of T1-weighted sequences enables the assessment of both anatomical features and lymph flow rate, yielding dynamic flow results [20]. This technique may be particularly valuable in cases such as uro-lymphatic fistula responsible for chyluria, where the search extends beyond the lymphatic system to identify lymph leaks.

Conventional lymphography, performed after locating the lymphatics of the dorsum of the foot by injecting methylene blue and slow injection of iodinated contrast medium, is rarely used but can still be useful to inform on the site, size, and number of uro-lymphatic communications prior to surgery [16, 21]. However, this technique is operator-dependent, invasive, and contraindicated in cases of lower limb lymphedema. Foot injection may be replaced by lymph node injection.

In 2018, Dong et al. [22] described how they used CT within 60 min after conventional lymphography to identify lymphatic anomalies and the distribution of collateral lymphatic vessels. This technique has the same disadvantages as conventional lymphography.

Lymphangioscintigraphy is a functional imaging technique using, mainly, 99mTc sulfurmicrocolloid to explore chyluria and can demonstrate abnormal lymphatic drainage. However, the spatial resolution remains suboptimal [23].

## Therapeutic implications

Management is based on identifying the cause of chyluria and its specific pattern and depends on the severity of the chyluria and presence of associated symptoms. MR lymphography is of prime importance to determine the specific mechanism of uro-lymphatic fistula.

Conservative management including a low fat diet with a high fluid intake has a success rate of more than 70% [24]. For the patients with suspected filarial infection, medication is used in combination with dietary modifications. Diethlycarbamazine, ivermectin and albendazole are the most commonly used medications [2].

The most commonly used minimally invasive techniques is sclerotherapy that is indicated in patients that have failed conservative or medical treatments. Sclerotherapy, when instilled into the urinary tract, reaches the lymphatics via the uro-lymphatic fistula. It then induces a chemical lymphangitis with the edema causing blockage of lymphatics and resulting in relief [25].

Invasive management of chyluria is reserved for those with major symptoms and in whom medical or minimally invasive treatment options have failed. The operative techniques described for chyluria include uro-lymphatic disconnection and creation of lymphovenous anastomoses. For these patients evaluation with MR lymphography is very important. Indeed, it is necessary to specify if the site of uro-lymphatic fistula is single or multiple and if there are diffuse anomalies of the lymphatic system. When the uro-lymphatic fistula is localized to a single site, uro-lymphatic disconnection appears to be the optimal solution. However, in cases where the anomalies are widespread, lymphovenous anastomoses are preferred [26].

## Conclusion

This pictorial review shows how non-enhanced MR lymphography based on heavily T2-weighted fast spin-echo sequences and MIP reconstructions provides a non-invasive, non-irradiating, and well-detailed imaging modality for patients presenting with chyluria. This technique allows radiologists to evaluate the lymphatic system and its numerous anatomic variations, to map one or several lymphatic anomalies, and to precisely locate a urolymphatic fistula. Additional research can be dedicated to optimizing the non-enhanced MR lymphography technique, with a focus on enhancing image quality, resolution, and acquisition time. This endeavor may involve exploring advanced pulse sequences, coil designs, and post-processing methods to improve the visualization of lymphatic vessels and urinary tract abnormalities. Moreover, conducting comparative studies to assess the diagnostic performance of non-enhanced MR lymphography in comparison with other imaging modalities, particularly gadolinium-enhanced MR lymphangiography, would be valuable. Such studies would help refine the analysis of the clinical usefulness of MR lymphography in evaluating uro-lymphatic fistulae. These research perspectives have the potential to advance our understanding, diagnosis, and management of chyluria, ultimately resulting in enhanced patient outcomes.

## Data Availability

The datasets generated during and/or analyzed during the current study are available from the corresponding author on reasonable request.

## Abbreviations

MR: Magnetic Resonance
3D: Three-dimensional
MIP: Maximum Intensity Projection
GLA: Generalized lymphatic anomaly
